# Effects of nicotinamide and carbogen on tumour oxygenation, blood flow, energetics and blood glucose levels

**DOI:** 10.1054/bjoc.2000.1144

**Published:** 2000-05-18

**Authors:** S P Robinson, F A Howe, M Stubbs, J R Griffiths

**Affiliations:** CRC Biomedical Magnetic Resonance Research Group, Division of Biochemistry, St. George's Hospital Medical School, Cranmer Terrace, London, SW17 0RE, UK

**Keywords:** carbogen, nicotinamide, hydralazine, oxygenation, blood flow, MRI

## Abstract

Both host carbogen (95% oxygen/5% carbon dioxide) breathing and nicotinamide administration enhance tumour radiotherapeutic response and are being re-evaluated in the clinic. Non-invasive magnetic resonance imaging (MRI) and ^31^P magnetic resonance spectroscopy (MRS) methods have been used to give information on the effects of nicotinamide alone and in combination with host carbogen breathing on transplanted rat GH3 prolactinomas. Gradient recalled echo (GRE) MRI, sensitive to blood oxygenation changes, and spin echo (SE) MRI, sensitive to perfusion/flow, showed large signal intensity increases with carbogen breathing. Nicotinamide, thought to act by suppressing the transient closure of small blood vessels that cause intermittent tumour hypoxia, induced a small increase in blood oxygenation but no detectable change in perfusion/flow. Carbogen combined with nicotinamide was no more effective than carbogen alone. Both carbogen and nicotinamide caused significant increases in the nucleoside triphosphate/inorganic phosphate (βNTP/P _i_) ratio, implying that the tumour cells normally receive sub-optimal substrate supply, and is consistent with either increased glycolysis and/or a switch to more oxidative metabolism. The most striking observation was the marked increase in blood glucose (twofold) induced by both nicotinamide and carbogen. Whether this may play a role in tumour radiosensitivity has yet to be determined. Copyright 2000 Cancer Research Campaign© 2000 Cancer Research Campaign


British Journal of Cancer (2000) 82(12), 2007–2014
© 2000 Cancer Research Campaign
doi: 10.1054/ bjoc.2000.1144, available online at http://www.idealibrary.com on IDEAL®

### Effects of nicotinamide and carbogen on tumour oxygenation, blood flow, energetics and blood glucose levels

**SP Robinson, FA Howe, M Stubbs and JR Griffiths**

CRC Biomedical Magnetic Resonance Research Group, Division of Biochemistry, St. George's Hospital Medical School, Cranmer Terrace, London SW17 0RE, UK

**Summary** Both host carbogen (95% oxygen/5% carbon dioxide) breathing and nicotinamide administration enhance tumour radiotherapeutic response and are being re-evaluated in the clinic. Non-invasive magnetic resonance imaging (MRI) and $^{31}$P magnetic resonance spectroscopy (MRS) methods have been used to give information on the effects of nicotinamide alone and in combination with host carbogen breathing on transplanted rat GH3 prolactinomas. Gradient recalled echo (GRE) MRI, sensitive to blood oxygenation changes, and spin echo (SE) MRI, sensitive to perfusion/flow, showed large signal intensity increases with carbogen breathing. Nicotinamide, thought to act by suppressing the transient closure of small blood vessels that cause intermittent tumour hypoxia, induced a small increase in blood oxygenation but no detectable change in perfusion/flow. Carbogen combined with nicotinamide was no more effective than carbogen alone. Both carbogen and nicotinamide caused significant increases in the nucleoside triphosphate/inorganic phosphate (βNTP/$P_i$) ratio, implying that the tumour cells normally receive sub-optimal substrate supply, and is consistent with either increased glycolysis and/or a switch to more oxidative metabolism. The most striking observation was the marked increase in blood glucose (twofold) induced by both nicotinamide and carbogen. Whether this may play a role in tumour radiosensitivity has yet to be determined. © 2000 Cancer Research Campaign

**Keywords:** carbogen; nicotinamide; hydralazine; oxygenation; blood flow; MRI

Tumour oxygenation and blood flow are of fundamental importance to many forms of cancer therapy. Poorly-perfused regions of tumours are likely to be hypoxic and thus resistant to radiotherapy (Gray et al, 1956). At present it is believed that in addition to the chronic, diffusion-limited hypoxia described by Thomlinson and Gray (1955), there is a second mechanism – transient, acute hypoxia in small (50 μm diameter) tumour volumes (Chaplin et al, 1987; Braun et al, 1999). Both nicotinamide and carbogen (95% oxygen/5% carbon dioxide) have been shown to increase tumour response to radiotherapy (Horsman et al, 1987; Chaplin et al, 1991; Kjellen et al, 1991), and it is generally considered that they target these two different hypoxia mechanisms. Breathing carbogen increases the amount of dissolved oxygen in the plasma at the capillary level and this, assisted by hypercapnic-induced vasodilation, may allow diffusion of oxygen into chronically hypoxic regions of tumours, resulting in an increase in tumour oxygenation. Nicotinamide is thought to reduce the occurrence of acute hypoxia (Chaplin et al, 1990) and hence increase tumour blood flow (Horsman et al, 1988; Hirst et al, 1993), although its precise mechanism of action is still unclear. The combination of carbogen breathing and nicotinamide is currently being re-evaluated in the clinic as a strategy to overcome hypoxic cell radioresistance (Hoskin et al, 1997; Kaanders et al, 1998; Bernier et al, 1999).

*Received 30 November 1999*
*Revised 29 January 2000*
*Accepted 5 February 2000*

*Correspondence to:* SP Robinson

The response of tumours to host carbogen breathing has been successfully monitored by $^1$H MRI methods with high temporal and spatial resolution, and which are sensitive to the deoxyhaemoglobin concentration. Deoxyhaemoglobin is paramagnetic and its presence creates inhomogeneities in the magnetic field. This reduces the $T_2$* magnetic resonance (MR) relaxation time of the tissue surrounding blood vessels containing deoxygenated blood. Gradient-recalled echo (GRE) images are sensitive to $T_2$*, thus a change in GRE image intensity reflects a change in blood deoxygenation due to either a change in blood saturation or blood flow. Deoxyhaemoglobin therefore acts as an endogenous, blood oxygenation level dependent (BOLD) contrast agent (Ogawa et al, 1990). GRE MR images are also sensitive to the so-called 'in-flow effect' whereby the water in fresh blood flowing into the selected imaging slice is not saturated from the previous radiofrequency pulse, thus giving a stronger signal than that from static water in tissue (Duyn et al, 1994). Several studies have demonstrated large carbogen-induced increases in $T_2$* in both rodent (Robinson et al, 1995; Dunn and Swartz, 1997; Oikawa et al, 1997; Robinson et al, 1997, 1999) and human (Griffiths et al, 1997) tumours. This is a consequence of an improvement in both tumour blood flow and oxygenation (Howe et al, 1996; Al-Hallaq et al, 1998), a method subsequently termed FLOOD (*Flo*w and *O*xygen *D*ependent) imaging (Howe et al, 1999).

In preclinical in vivo studies, sensitization is only seen when nicotinamide is administered prior to radiotherapy (Horsman, 1995), with an apparent maximum observed when given ca. 1 hour prior to treatment (Horsman et al, 1987). To try and elucidate the mechanisms behind nicotinamide-induced tumour radiosensitization, the temporal response of rat GH3 prolactinomas to nicotinamide, alone and in combination with carbogen, was monitored by three MR methods. GRE MR imaging was used to monitor blood oxygenation via $T_2$*; spin echo (SE) MR imaging to monitor flow via the changes in the $T_1$* relaxation time (Howe et al, 1999); and $^{31}$P MRS to detect changes in tumour bioenergetics (e.g. βNTP/$P_i$ ratio) (Tozer and Griffiths, 1992). The combination of these MR methods was firstly validated in a pilot study following the response of GH3 prolactinomas to hydralazine, a vasodilator whose tumour vascular steal-effects are well documented (Jirtle, 1988; Robinson et al, 1998). Subsequently the tumour response to nicotinamide and carbogen was studied in vivo using MR and other complementary methods to elucidate the underlying mechanisms of action.

### MATERIALS AND METHODS

### Animals and tumours

GH3 prolactinomas were grown in the flanks of female Wistar Furth rats. Tumour cells from a serial passage of a cell suspension (Prysor-Jones and Jenkins, 1981) were injected subcutaneously into 180–200 g rats and tumours grown to 1.5–2 cm diameter.

Anaesthesia was induced with a 4 ml $kg^{-1}$ intraperitoneal injection of fentanyl citrate (0.315 mg $ml^{-1}$) plus fluanisone (10 mg $ml^{-1}$) ('Hypnorm', Janssen Pharmaceutical Ltd), midazolam (5 mg $ml^{-1}$) ('Hypnovel', Roche) and water (1:1:2). This combination has a minimal effect on tumour blood flow (Menke and Vaupel, 1988) and $^{31}$P MRS characteristics (Sansom and Wood, 1994). The tail vein was cannulated prior to MR, to allow administration of hydralazine (Sigma, UK) or nicotinamide (Sigma, UK) whilst the animal remained in the magnet bore. The animals were placed on a flask containing circulating warm water to maintain the core temperature at 37°C and positioned so the tumour hung vertically into a radiofrequency coil. Carbogen (BOC, UK Ltd) was administered via a nose-piece, equipped with a scavenger to prevent the leakage of paramagnetic oxygen into the magnet bore, which could potentially change the magnetic susceptibility around the coil and produce image artefacts (Bates et al, 1995).

### MRI and MRS

$^{1}$H MRI and $^{31}$P MRS was performed with a 4.7 T, 33 cm SISCO (Spectroscopy Imaging Systems Corporation) instrument fitted with a 10 G $cm^{-1}$, 12-cm bore high-performance auxiliary gradient insert, using a two-turn 3-cm coil tuneable to both $^{1}$H and $^{31}$P resonant frequencies. Prior to data acquisition, field homogeneity was optimized by shimming on the water signal for each tumour to a linewidth of between 50 and 70 Hz. GRE images (echo time TE = 20 ms, repetition time TR = 80 ms, flip angle $\alpha$ = 45°) and SE images (TE = 20 ms, TR = 300 ms) were acquired from a single 1 mm slice taken through the centre of the tumour. Each image took 3 min to acquire using 256 phase encode steps over a 4 cm field-of-view (FOV) with 8 averages. Non-localized $^{31}$P spectra were acquired using a hard pulse with TR = 3 s, 64 transients and an acquisition time of 4 min. The hard pulse flip angle was optimized to minimize the appearance of PCr from surrounding muscle tissue.

Interleaved MRI and MRS were acquired from separate cohorts ($n$ = 6) of tumours for an initial 20 min of baseline (air breathing with no vasoactive agent) data, and thereafter using one of the following protocols.

1. 5 mg $kg^{-1}$ of hydralazine administered with air breathing for 40 min
2. 1000 mg $kg^{-1}$ nicotinamide in saline administered followed by 70 min air breathing
3. initial 20 min carbogen breathing alone, resumption of air breathing for 40 min with administration of 1000 mg $kg^{-1}$ nicotinamide, and finally 30 min carbogen breathing.

### MR data analysis

For the images, a region of interest (ROI) encompassing the whole tumour $^{1}$H image but excluding the skin was chosen and the average pixel intensity calculated. Image intensities are reported relative to the average pixel intensity in the ROI during initial air breathing which was set to 100%.

Spectral analysis was performed using the Variable Projection (VARPRO) time-domain non-linear least squares method (van den Boogaart et al, 1995). For each analysis the first three data points were excluded from the fit to eliminate the influence of fast decaying signals from immobilized phosphates which cause a baseline hump in the spectra. The data were fitted assuming contributions from phosphomonoesters (PME), inorganic phosphate ($P_i$), phosphodiesters (PDE), phosphocreatine (PCr) and the three nucleoside triphosphates (NTP) resonances, and peak lineshape was assumed to be Lorentzian. Peak area ratios of βNTP/$P_i$ and $P_i/\Sigma P$ were then determined. Intracellular tumour $pH_i$ was determined using the VARPRO-derived chemical shifts for the $P_i$ and $\alpha$-NTP resonances (Ojugo et al, 1999).

### Blood pressure monitoring

Mean arterial blood pressure (MABP) was measured over the same time course as for the MR protocols on separate cohorts of rats ($n$ = 5), using a rat tail blood pressure monitor (Harvard Apparatus Ltd, Edenbridge, UK).

### Blood plasma glucose

Arterial blood samples were taken from the iliac artery of a separate cohort of tumour-bearing rats before and (1) 40 min post-administration of 1000 mg $kg^{-1}$ nicotinamide intravenously or (2) after 10 min of carbogen breathing ($n$ = 10 samples per treatment group). The blood samples were centrifuged to remove the red cells, an aliquot of the plasma supernatant was deproteinized with perchloric acid and subsequently neutralized. Glucose was determined on the neutralized extracts according to Bergmeyer (1974).

### Statistical analysis

The reproducibility of the MRI and $^{31}$P MRS acquisitions was assessed from the two sets of pre-challenge measurements made in each protocol. For the normalized GRE and SE image intensities, βNTP/$P_i$ and $P_i/\Sigma P$, the coefficient of variation (CV) was measured in each of the 18 animals and the r.m.s. value determined. For $pH_i$, the standard deviation was measured and the r.m.s. determined. Results are presented as mean ± standard error, and significant changes identified using Student's two-tailed $t$-test at a 5% confidence level.

© 2000 Cancer Research Campaign

### RESULTS

In all the studies the blood-oxygenation-sensitive GRE images showed a heterogeneous pattern of intensities whereas the flow-sensitive SE images showed a fairly homogeneous pattern. In the GRE images during air breathing, the regions of high signal intensity are thought to delineate well-oxygenated/perfused areas of the tumour, whilst dark areas are thought to indicate poorly perfused/necrotic regions. The small hyperintense spots in both SE and GRE images are probably attributable to signal from large blood vessels (Howe et al, 1999). In the $^{31}$P MR spectra, typical resonances were identified for PME, $P_i$, PDE, PCr and γ, α and β-NTP. Non-localized $^{31}$P MRS was utilized to maintain adequate temporal resolution and can result in spectral contamination. However, in all the acquired spectra the PCr peak, when present, was always less than that of NTP.

In the pilot study, hydralazine produced the expected significant *decreases* in both GRE and SE image intensity and in $\beta NTP/P_i$ after 5 min. After 20 min the changes were maximal and stable for the further 20 min of measurements. Within some of the GRE and SE images, bright structures were observed which *decreased* in number and intensity post-hydralazine (Figure 1).

Figure 2 shows representative GRE and SE MR images and $^{31}$P spectra from a GH3 prolactinoma where the changes following nicotinamide challenge had reached a maximum. Figure 3 shows the time course of changes in MR image intensity and $^{31}$P MRS parameters following administration of nicotinamide. A significant increase in $\beta NTP/P_i$ was observed 10 min after administration of nicotinamide; the maximal increase was reached after 40 min and it was then stable for a further 30 min. Concurrent with this was a significant decrease in $P_i/\Sigma P$ and a small but statistically non-significant increase in tumour $pH_i$. Changes in the oxygenation-sensitive average GRE MR image intensity over the tumour were much less but there was a small significant signal increase after 40 min. The SE MR images, which are sensitive to blood flow, showed no change in average image intensity.

These results formed the basis of the protocol designed to assess the combination of carbogen and nicotinamide; carbogen breathing was started 40 min post-nicotinamide when the maximum response to nicotinamide occurred. The response to carbogen breathing alone was much greater and faster than that with nicotinamide alone. Significant increases in both GRE and SE image intensity and in $\beta NTP/P_i$ were observed after 5 min of carbogen breathing with maximum increases after 10 min. Figure 4 shows representative GRE and SE MR images of the maximum response to host carbogen breathing. On return to air-breathing these changes were reversed within 5 min. When carbogen was given 40 min after administration of nicotinamide, the $^{1}$H MRI and $^{31}$P MRS changes were no different to those caused by carbogen breathing alone. Hyperintensities in both GRE and SE images increased in number and intensity with carbogen breathing, irrespective of whether nicotinamide had been administered (Figure 4).

Table 1 summarizes the data for each vascular challenge when MRI and MRS changes were maximal and stable, i.e. 40 min after hydralazine administration, 40 min after nicotinamide administration and after 10 min of carbogen breathing. The data during air breathing represent the average of data from all three of the previously described protocols, but prior to the vascular challenge. From the two successive MRI and $^{31}$P MRS measurements in all 18 animals prior to treatment, the precision of the measurements was determined: these were 3% for GRE MRI intensity, 2% for SE MRI intensity, 23% for $\beta NTP/P_i$, 19% for $P_i/\Sigma P$ (all r.m.s. CV) and 0.1 units for pH (r.m.s. std. dev.).

Mean arterial blood pressure was unchanged by nicotinamide and carbogen but significantly reduced by hydralazine (Table 1).

Circulating blood glucose levels were determined prior to and either 40 min post-administration of nicotinamide or after 10 min of carbogen breathing, these time points selected on the basis of the maximum observed improvement in tumour energetics. Both nicotinamide ($11.4 \pm 0.7$ μmol ml$^{-1}$) and carbogen breathing ($15.6 \pm 0.6$ μmol ml$^{-1}$) induced significant increases in plasma glucose levels (Table 1). The control plasma glucose levels ($6.6 \pm 0.3$ μmol ml$^{-1}$) and the enhanced levels after carbogen breathing were similar to those previously reported (Stubbs et al, 1998).

### DISCUSSION

The observed MRI and MRS responses of GH3 prolactinomas to hydralazine were as expected, and this pilot study validated our interpretation of the changes seen with nicotinamide and carbogen. Hydralazine acts directly on vascular smooth muscle in vessels of normal tissues, causing vasodilation and an overall decrease in MABP. Tumour blood vessels, which may lack smooth muscle, do not dilate in response to hydralazine, resulting in a redistribution of blood away from the tumour, described as vascular steal (Jirtle, 1988), and hence a reduction in tumour blood flow. This reduction in tumour perfusion results in nutrient and oxygen deprivation, and hence reduced bioenergetic status as observed in the $^{31}$P MRS spectrum (an increase in $P_i$ relative to NTP). This has also been observed for hydralazine in other tumour models (Okunieff et al, 1988; Dunn et al, 1989; Bhujwalla et al, 1990; Robinson et al, 1998). SE MR images (Figure 1 C,D) are sensitive to flow, and hydralazine causes a decrease in overall signal intensity due to reduced perfusion. The hyperintense spots are from the water in blood vessels and are thus identified as large blood vessels in cross-section. This is confirmed by their reduction in number in response to hydralazine, the reduced perfusion resulting in less of an 'in-flow' effect. The overall reduction in GRE image signal intensity reflects the increase in capillary blood deoxyhaemoglobin as the reduced perfusion means a larger oxygen fraction is extracted. A similar GRE MRI response to hydralazine has been observed in RIF-1 fibrosarcomas (Bhujwalla et al, 1994; Williams et al, 1996).

Despite the plethora of data demonstrating the ability of nicotinamide to radiosensitize (Chaplin et al, 1991; Kjellen et al, 1991; Horsman 1995 and references therein), there appears to be no consensus on its precise mechanism of action. The main aim of this study was to investigate tumour response to nicotinamide administration and carbogen inhalation, which were given separately and in combination. Carbogen caused marked and widespread increases ($39 \pm 2\%$) in GRE MR image intensity, whereas those caused by nicotinamide were much smaller ($8 \pm 3\%$), though still statistically significant (Table 1). The results with carbogen were qualitatively similar to those seen in our previous studies on this tumour model which we interpreted as largely due to decreased deoxyhaemoglobin in the tumour blood vessels (Robinson et al, 1995, 1997, 1999; Howe et al, 1996, 1999). It should be noted that the GRE MR images with short TRs are also susceptible to in-flow effects, and hence an increase in blood flow

© 2000 Cancer Research Campaign

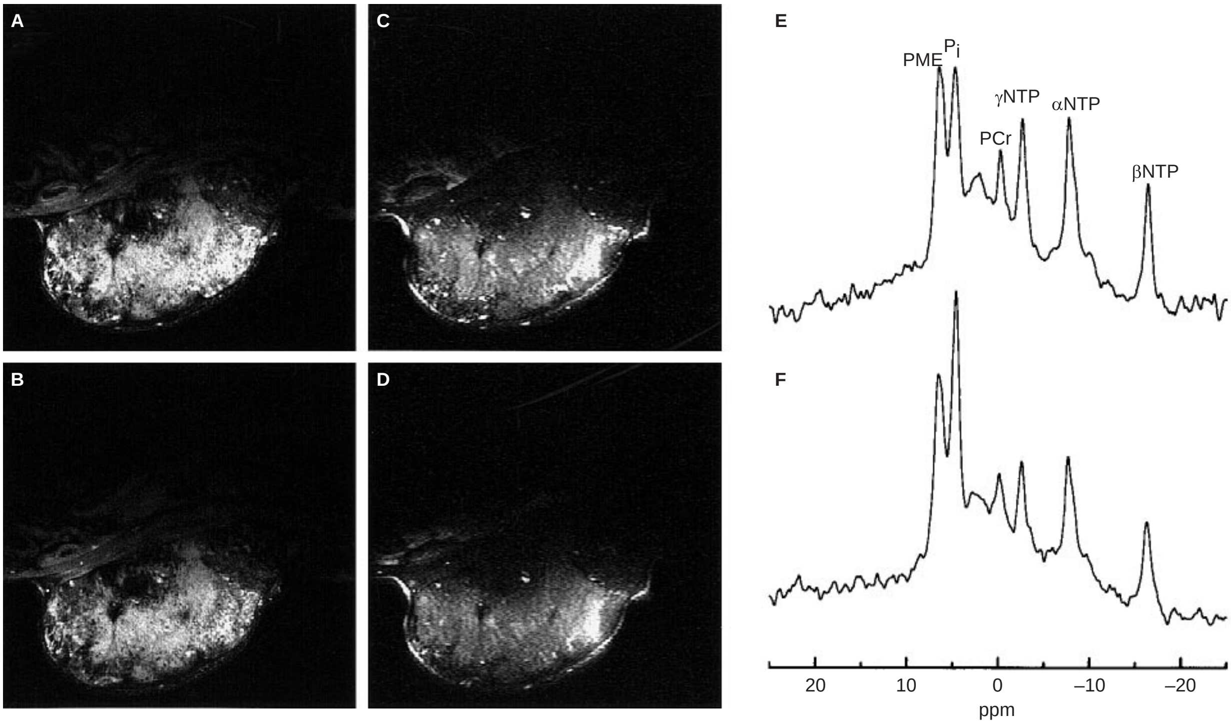

**Figure 1** Response of a GH3 prolactinoma to 5 mg kg$^{-1}$ hydralazine i.v., monitored by interleaved $^{1}$H MRI & $^{31}$P MRS: (**A** and **B**) are GRE MR images prior to and 32 min post-hydralazine; (**C** and **D**) are SE MR images prior to and 35 min post-hydralazine; (**E** and **F**) are non-localized $^{31}$P MR spectra prior to and 38 min post-hydralazine

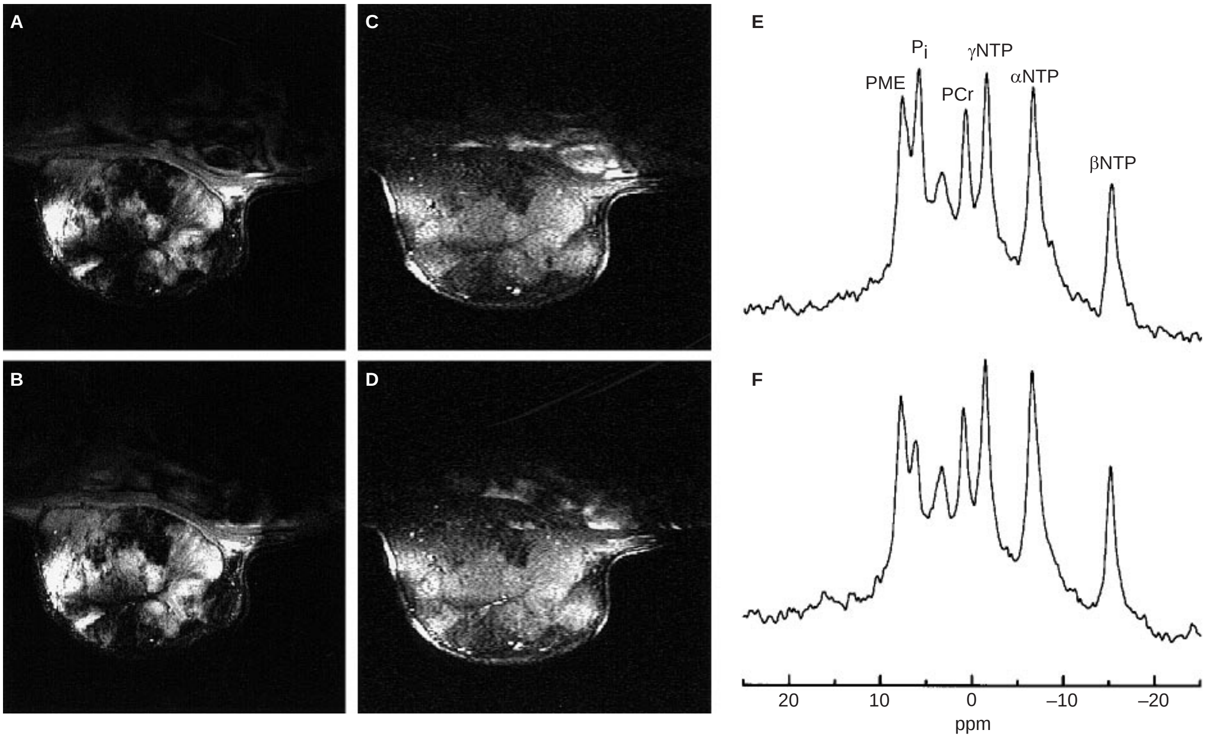

**Figure 2** Response of a GH3 prolactinoma to 1000 mg kg$^{-1}$ nicotinamide administered i.v., monitored by interleaved $^{1}$H MRI & $^{31}$P MRS: (**A** and **B**) are GRE MR images prior to and 42 min post-nicotinamide; (**C** and **D**) are SE MR images prior to and 45 min post-nicotinamide; (**E** and **F**) are non-localized $^{31}$P MR spectra prior to and 48 min post-nicotinamide

*© 2000 Cancer Research Campaign*

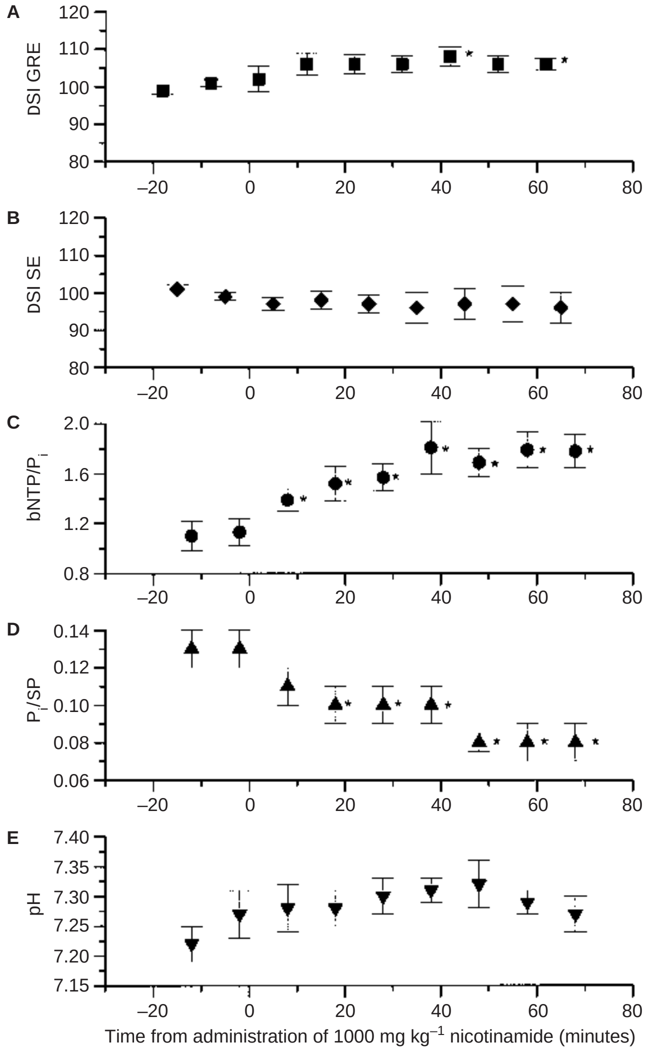

**Figure 3** Time course of $^{1}$H MR imaging and $^{31}$P MR spectroscopy changes prior to and following administration of 1000 mg kg$^{-1}$ nicotinamide. (**A**) Normalized GRE MR image intensity (%). (**B**) Normalized SE MR image intensity (%). (**C**) $\beta$NTP/$P_i$ (**D**) $P_i/\Sigma P$. (**E**) $pH_i$. All data are mean ± s.e.m. for $n = 6$. *$P < 0.05$ Student's two-tailed *t*-test could also result in an increase in GRE signal (Duyn et al, 1994). A secondary effect that we demonstrated herein with SE MRI was enhanced blood flow into vessels within the tumour slice being imaged, and here again the results with carbogen in the present study were qualitatively similar to those we have previously published (Howe et al, 1999). Nicotinamide, however, had no effect on the SE images, suggesting that it did not cause changes in blood flow in the tumour vessels that we were able to image. Flow-sensitive MRI perfusion maps of 9L rat brain gliomas also showed no change in response to nicotinamide (Brown et al, 1999).

In general, these results can be understood in terms of the accepted mechanisms of action of carbogen and nicotinamide. Since carbogen inhalation causes vasodilation (because of the hypercapnia) and enhanced oxygen transport (because of the hyperoxia) it is not surprising that there is evidence of enhanced blood flow and decreased vascular deoxyhaemoglobin content of the tumours. Furthermore, these effects are likely to be widespread throughout the tumour, so the overall signal intensity of the image is likely to change. Nicotinamide, on the other hand, is thought to act by suppressing the transient closure of small blood vessels that causes intermittent tumour hypoxia (Chaplin et al, 1987). Studies using window chamber tumours (Eddy and Cassarett, 1973; Yamaura and Matsuzawa, 1979; Dewhirst et al, 1992) or histological methods (Chaplin et al, 1987) have indicated that less than 10% of tumour blood vessels are subject to intermittent hypoxia at any one time. This would be consistent with the present results in which nicotinamide changed the GRE image intensity by only 8%. In a study by Kimura et al (1996) up to 30% of the tissue in a mammary tumour model was found to contain vessels subject to unstable blood flow and thus liable to experience transient hypoxia, but the volume of transiently hypoxic tissue at any one time was not calculated. If each susceptible vessel were closed for 30% of the time the overall volume of transiently hypoxic tumour tissue would still be about 10%. Our SE MRI experiments directly addressed the question of tumour blood flow, and we found that nicotinamide had no effect on flow into the imaged slice. This, however, is explicable, since the vessels we are able to image are quite large (> 0.3 mm), whereas the transient hypoxia phenomenon occurs in vessels of less than 0.1 mm diameter (Kimura et al, 1996).

When carbogen and nicotinamide were administered sequentially, the addition of nicotinamide made no significant difference to the GRE MRI image, i.e. carbogen followed by nicotinamide had the same effect as carbogen alone. This, too, is explicable in terms of the standard mechanisms of action of the two agents. There is no reason to think that their effects would be synergistic, and if they are additive one would not expect to be able to distinguish the small effect of nicotinamide superimposed on the larger one caused by carbogen. In radiobiological experiments, carbogen and nicotinamide in combination cause more radiosensitization than either treatment alone (Chaplin et al, 1991; Kjellen et al, 1991). The difference between this result and the present one could be due to the much smaller proportion of cells in a tumour that are radiobiologically hypoxic. Nicotinamide could have a major effect on radiobiological hypoxia by oxygenating some of these cells without significantly affecting the overall MRI response of the tumour.

The increased $\beta$NTP/$P_i$ ratio in response to carbogen in these GH3 tumours is unsurprising (although not all tumour models show such rises after carbogen challenge), if we assume that the tumour's oxygen supply is sub-optimal when the host is breathing air. If there are substantial, chronically hypoxic volumes of tissue then the improved blood flow and blood oxygen content caused by carbogen inhalation would be expected to enhance tumour energetics. In contrast, if the action of nicotinamide is confined to a small fraction of the cells in the tumour one would not expect to see such marked changes in the $\beta$NTP/$P_i$ ratio. A similar response has been previously reported in both SCCVII and KHT murine tumours (Wood et al, 1991), However, there is another factor to be taken into account: surprisingly, both these very different treatments caused marked and statistically significant hyperglycaemia.

We can explain the improved bioenergetic parameters in GH3 tumour in response to carbogen if we assume that the tumour cells normally receive sub-optimal substrate supply. Many studies with perfused tumours have shown that glucose consumption varies directly with glucose supply (Sauer et al, 1982; Vaupel et al, 1989).

*© 2000 Cancer Research Campaign*

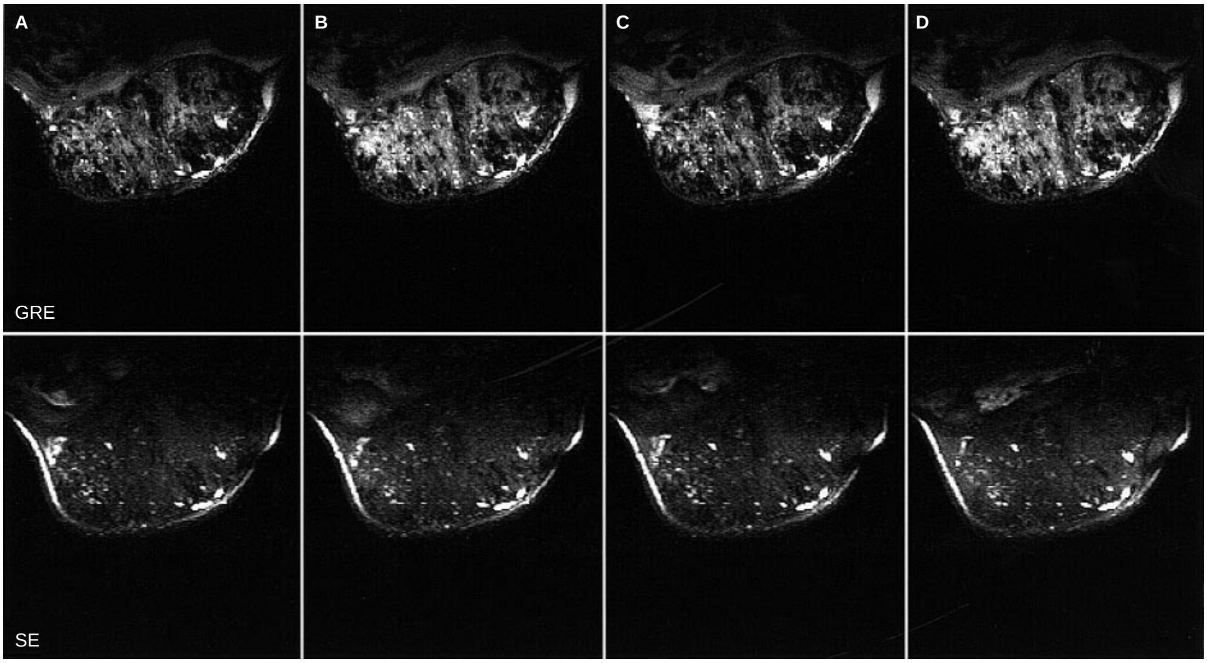

**Figure 4** GRE and SE $^1$H MR images of one GH3 prolactinoma acquired during (**A**) initial air breathing, (**B**) host carbogen breathing, (**C**) resumed air breathing and ca. 40 min post-administration of 1000 mg $kg^{-1}$ nicotinamide i.v. and (**D**) subsequent carbogen breathing and ca. 70 min post-nicotinamide

**Table 1**

| | Air | Hydralazine | Nicotinamide | Carbogen | Nicotinamide and carbogen |
|---|---|---|---|---|---|
| GRE SI | 100 | 85 ± 2[a] | 108 ± 3[b] | 139 ± 2[a] | 146 ± 5[a] |
| SE SI | 100 | 90 ± 2[a] | 100 ± 4 | 115 ± 2[a] | 117 ± 3[a] |
| $\beta$NTP/$P_i$ | 1.06 ± 0.02 | 0.66 ± 0.06[a] | 1.81 ± 0.21[a] | 1.58 ± 0.1[a] | 1.62 ± 0.14[a] |
| $P_i/\Sigma P$ | 0.13 ± 0.01 | 0.17 ± 0.01[a] | 0.08 ± 0.01[a] | 0.09 ± 0.01[a] | 0.09 ± 0.01[a] |
| pH | 7.22 ± 0.01 | 6.92 ± 0.04[a] | 7.32 ± 0.04 | 7.23 ± 0.02 | 7.26 ± 0.02 |
| MABP (mmHg) | 103 ± 6 | 46 ± 2[a] | 92 ± 7 | 112 ± 5 | 95 ± 4 |
| Glucose ($\mu$mol $ml^{-1}$) | 6.6 ± 0.3 | – | 11.4 ± 0.7[a] | 15.6 ± 0.6[a] | – |

[a]$P < 0.01$ compared to air. [b]$P < 0.05$ compared to air. Summary of the data for each vascular challenge when MRI and MRS changes were maximal and stable. The data during air breathing are the average of data from all three protocols prior to the vascular challenge.

Since carbogen and nicotinamide cause approximately doubled blood glucose concentrations, it is not, therefore, surprising that they both enhance the tumour $\beta$NTP/$P_i$ ratio. It is not possible to deduce whether the glucose substrate in the present experiments was metabolized oxidatively or glycolytically, and there are reports of both types of metabolism in the literature. Dewhirst et al (1999) showed that combined hyperglycaemia and hyperoxia improved tumour $pO_2$ more than hyperoxia alone, suggesting that the R3230Ac tumour line they studied switched from an oxidative to a more glycolytic metabolism when challenged with glucose, thus sparing oxygen – a Crabtree effect. However, in $^{13}$C MRS dynamic studies in the RIF-1 tumour, Nielsen et al (1999) have shown that carbogen breathing significantly *decreases* the 'apparent' glycolytic (i.e. $^{13}$C glucose to $^{13}$C lactate) rate, suggesting a more oxidative metabolism. Similarly Stubbs et al (1998) showed carbogen-induced hyperglycaemia accompanied by a decrease in [lactate] (in Morris hepatoma 9618a), also consistent with a switch to a more oxidative metabolism. Despite these differing observations of the metabolic fate of glucose, they are all consistent with enhanced energetic status in response to an increased substrate supply.

In summary, the MRI results can be accounted for on the basis of the accepted mechanisms of action of carbogen and nicotinamide, whereas the $^{31}$P MRS changes can be explained by the raised (~twofold) blood glucose induced by these two agents. Systemic effects of raised blood glucose induced by nicotinamide and carbogen do not appear to have been considered in the literature with respect to tumour radiosensitization, although attempts to increase tumour $pO_2$ by decreasing the consumption of oxygen, and hence radioresponse, have been (Biaglow et al, 1998). It has been known for many years that metabolism of nicotinamide results in glycogen breakdown and a consequent increase in blood glucose (Ammon and Estler, 1967; Moreno et al, 1985). However, we have not found any previous reports (other than our own work, Stubbs et al, 1998) of carbogen-induced hyperglycaemia and the mechanism of this effect must be speculative. Carbogen breathing

 © 2000 Cancer Research Campaign induces hypercapnia which is known to cause an excitatory response of the sympathetic nervous system and epinephrine release. Epinephrine induces glycogenolysis as well as stimulation of cardiac output and metabolic rate via the adrenal medulla (Guyton and Hall, 1996). The impact of these systemic effects on tumour physiology and metabolism is clearly complex and may well influence how a tumour responds to radiotherapy in the presence of clinical radiosensitizers. High levels of hyperglycaemia induced by glucose infusion (fourfold higher than normal blood glucose) have been shown to decrease tumour blood flow and pH and used as an adjuvant for hyperthermia (Song, 1998 and therein) but these effects probably do not play a role in this study in which the degree of hyperglycaemia was much less severe. However, in view of the current clinical radiotherapy trials of combined nicotinamide and carbogen administration to patients, it would be prudent to check for hyperglycaemia in human subjects.

### ACKNOWLEDGEMENTS

This work was supported by the Cancer Research Campaign, UK, [CRC] grant SP 1971/0402 and SP 1971/0502. The authors would like to thank Loreta Rodrigues for technical assistance, and Chris Brown and his staff for care of the animals.

### REFERENCES

Al-Hallaq HA, River JN, Zamora M, Oikawa H and Karczmar GS (1998) Correlation of magnetic resonance and oxygen microelectrode measurements of carbogen-induced changes in tumor oxygenation. *Int J Radiat Oncol Biol Phys* **41**: 151–159

Ammon HPT and Estler CJ (1967) The effect of nicotinic acid on glycolytic carbohydrate breakdown in the liver. *Life Sci* **6**: 641–647

Bates S, Yetkin Z, Jesmanowicz A, Hyde JS, Bandettini PA, Estkowski L and Haughton VM (1995) Artifacts in functional magnetic resonance imaging from gaseous oxygen. *J Magn Reson Imaging* **5**: 443–445

Bergmeyer HU (1974) *Methods of Enzymatic Analysis*. Verlag Chemie: Weinheim

Bernier J, Denekamp J, Rojas A, Trovo M, Horiot JC, Hamers H, Antognoni P, Dahl O, Richaud P, Kaanders J, van Glabbeke M and Pierart M (1999) ARCON: accelerated radiotherapy with carbogen and nicotinamide in non small cell lung cancer: a phase I/II study by the EORTC. *Radiother Oncol* **52**: 149–156

Bhujwalla ZM, Tozer GM, Field SB, Maxwell RJ and Griffiths JR (1990) The energy metabolism of RIF-1 tumours following hydralazine. *Radiother Oncol* **19**: 281–291

Bhujwalla ZM, Shungu DC, He Q, Wehrle JP and Glickson JD (1994) MR studies of tumors: relationship between blood flow, metabolism and physiology. In: *NMR in Physiology and Biomedicine*, Gillies RJ (ed), pp. 311–328. Academic Press: San Diego

Biaglow JE, Manevich Y, Leeper D, Chance B, Dewhirst MW, Jenkins WT, Tuttle SW, Wroblewski K, Glickson JD, Stevens C and Evans SM (1998) MIBG inhibits respiration: potential for radio- and hyperthermic sensitization. *Int J Radiat Oncol Biol Phys* **42**: 871–876

Braun RD, Lanzen JL and Dewhirst MW (1999) Fourier analysis of fluctuations of oxygen tension and blood flow in R3230Ac tumors and muscle in rats. *Am J Physiol* **277**: H551–H568

Brown SL, Ewing JR, Kolozsvary A, Butt S, Cao Y and Kim JH (1999) Magnetic resonance imaging of perfusion in rat cerebral 9L tumor after nicotinamide administration. *Int J Radiat Oncol Biol Phys* **43**: 627–633

Chaplin DJ, Olive PL and Durand RE (1987) Intermittent blood flow in a murine tumor: radiobiological effects. *Cancer Res* **47**: 597–601

Chaplin DJ, Horsman MR and Trotter MJ (1990) Effect of nicotinamide on the microregional heterogeneity of oxygen delivery within a murine tumor. *J Natl Cancer Inst* **82**: 672–676

Chaplin DJ, Horsman MR and Aoki DS (1991) Nicotinamide, Fluosol DA and carbogen: a strategy to reoxygenate acutely and chronically hypoxic cells in vivo. *Br J Cancer* **63**: 109–113

Dewhirst MW, Vinuya RZ, Ong ET, Klitzman B, Rosner G, Secomb TW and Gross JF (1992) Effects of bradykinin on the hemodynamics of tumor and granulating normal tissue microvasculature. *Radiat Res* **130**: 345–354

Dewhirst MW, Snyder S, Lanzen J, Braun RD, Secomb TW and Biaglow J (1999) Hyperglycemia plus hyperoxia improves tumor oxygenation more efficiently than hyperoxia alone. *Proc Int Soc Oxygen Transport Tiss* 36

Dunn JF and Frostick S, Adams GE, Stratford IJ, Howells N, Hogan G and Radda GK (1989) Induction of tumour hypoxia by a vasoactive agent. A combined NMR and radiobiological study. *FEBS Lett* **249**: 343–347

Dunn JF and Swartz HM (1997) Blood oxygenation: heterogeneity of hypoxic tissues monitored using BOLD MR imaging. In: *Oxygen Transport in Tissue XIX*, Harrison and Delpy (eds), pp. 645–650. Plenum Press: New York

Duyn JH, Moonen CTW, van Yperen GE, de Boer RW and Luyten PR (1994) Inflow versus deoxyhaemoglobin effects in 'BOLD' functional MRI using gradient echoes at 1.5T. *NMR Biomed* **7**: 83–88

Eddy HA and Cassarett GW (1973) Development of the vascular system in the hamster malignant neurilemoma. *Microvasc Res* **6**: 63–82

Gray LH, Conger AD, Ebert M, Hornsey S and Scott OCA (1956) The concentration of oxygen dissolved in tissues at the time of irradiation as a factor in radiotherapy. *Br J Radiol* **26**: 638–648

Griffiths JR, Taylor NJ, Howe FA, Saunders MI, Robinson SP, Hoskin PJ, Powell MEB, Thoumine M, Caine LA and Baddeley H (1997) The response of human tumors to carbogen breathing, monitored by gradient-recalled echo magnetic resonance imaging. *Int J Radiat Oncol Biol Phys* **39**: 697–701

Guyton AC and Hall JE (1996) *Textbook of Medical Physiology* WB Saunders: Philadelphia

Hirst DG, Joiner B and Hirst VK (1993) Blood flow modification by nicotinamide and metoclopramide in mouse tumours growing in different sites. *Br J Cancer* **67**: 1–6

Horsman MR (1995) Nicotinamide and other benzamide analogs as agents for overcoming hypoxic cell radiation resistance in tumours. *Acta Oncol* **34**: 571–587

Horsman MR, Chaplin DJ and Brown JM (1987) Radiosensitization by nicotinamide in vivo: a greater enhancement of tumor damage compared to that of normal tissues. *Radiat Res* **109**: 479–489

Horsman MR, Brown JM, Hirst VK, Lemmon MJ, Wood PJ, Dunphy EP and Overgaard J (1988) Mechanism of action of the selective tumor radiosensitiser nicotinamide. *Int J Radiat Oncol Biol Phys* **15**: 685–690

Hoskin PJ, Saunders MI, Phillips H, Cladd H, Powell MEB, Goodchild K, Stratford MRL and Rojas A (1997) Carbogen and nicotinamide in the treatment of bladder cancer with radical radiotherapy. *Br J Cancer* **76**: 260–263

Howe FA, Robinson SP and Griffiths JR (1996) Modification of tumour perfusion and oxygenation monitored by gradient recalled echo MRI and $^{31}$P MRS. *NMR Biomed* **9**: 208–216

Howe FA, Robinson SP, Rodrigues LM and Griffiths JR (1999) Flow and oxygenation dependent (FLOOD) contrast MR imaging to monitor the response of rat tumours to carbogen breathing. *Magn Reson Imaging* **17**: 1307–1318

Jirtle R (1988) Chemical modification of tumour blood flow. *Int J Hyperthermia* **4**: 355–371

Kaanders JHAM, Pop LAM, Marres HAM, Liefers J, van den Hoogen FJA, van Daal WAJ and van der Kogel AJ (1998) Accelerated radiotherapy with carbogen and nicotinamide (ARCON) for laryngeal cancer. *Radiother Oncol* **48**: 115–122

Kimura H, Braun RD, Ong ET, Hsu R, Secomb TW, Papahadjopoulos D, Hong K and Dewhirst MW (1996) Fluctuations in red cell flux in tumor microvessels can lead to transient hypoxia and reoxygenation in tumor parenchyma. *Cancer Res* **56**: 5522–5528

Kjellen E, Joiner MC, Collier JM, Johns H and Rojas A (1991) A therapeutic benefit from combining normobaric carbogen or oxygen with nicotinamide in fractionated X-ray treatments. *Radiother Oncol* **22**: 81–91

Menke H and Vaupel P (1988) Effect of injectable or inhalational anesthetics and of neuroleptic, neuroleptanalgesic, and sedative agents on tumor blood flow. *Radiat Res* **114**: 64–76

Moreno FJ, Sanchez-Urrutia L, Medina JM, Sanchez-Medina F and Mayor F (1985) Stimulation of phosphoenolpyruvate carboxykinase (guanosine triphosphate) activity by low concentrations of circulating glucose in perfused rat liver. *Biochem J* **150**: 51–58

Nielsen FU, Horsman MR, Daugaard P, Stodkilde-Jorgensen H and Maxwell RJ (1999) Tumor selective in vivo $^{13}$C-CP NMR assessment of glycolytic rate under various oxygenation states. *Proc Intl Soc Magn Reson Med* **2**: 1361

Ogawa S, Lee T-M, Nayak AS and Glynn P (1990) Oxygenation-sensitive contrast in magnetic resonance image of rodent brain at high magnetic fields. *Magn Reson Med* **14**: 68–78

© 2000 Cancer Research Campaign

Oikawa H, Al-Hallaq HA, Lewis MZ, River JN, Kovar DA and Karczmar GS (1997) Spectroscopic imaging of the water resonance with short repetition time to study tumor response to hyperoxia. *Magn Reson Med* **38**: 27–32

Ojugo ASE, McSheehy PMJ, McIntyre DJO, McCoy CL, Stubbs M, Leach MO, Judson IR and Griffiths JR (1999) Measurement of the extracellular pH of solid tumours in mice by magnetic resonance spectroscopy: a comparison of exogenous $^{19}$F and $^{31}$P probes. *NMR Biomed* **12**: 495–504

Okunieff P, Kallinowski F, Vaupel P and Neuringer LJ (1988) Effects of hydralazine-induced vasodilation on the energy metabolism of murine tumors studied by in vivo $^{31}$P-nuclear magnetic resonance spectroscopy. *J Natl Cancer Inst* **80**: 745–750

Prysor-Jones RA and Jenkins JS (1981) Effect of bromocriptine on DNA synthesis, growth and hormone secretion of spontaneous pituitary tumours in the rat. *J Endocrinol* **88**: 463–469

Robinson SP, Howe FA and Griffiths JR (1995) Noninvasive monitoring of carbogen-induced changes in tumor blood flow and oxygenation by functional magnetic resonance imaging. *Int J Radiat Oncol Biol Phys* **33**: 855–859

Robinson SP, Rodrigues LM, Ojugo ASE, McSheehy PMJ, Howe FA and Griffiths JR (1997) The response to carbogen breathing in experimental tumour models monitored by gradient-recalled magnetic resonance imaging. *Br J Cancer* **75**: 1000–1006

Robinson SP, van den Boogaart A, Maxwell RJ, Griffiths JR, Hamilton E and Waterton JC (1998) $^{31}$P-magnetic resonance spectroscopy and $^{2}$H-magnetic resonance imaging studies of a panel of early-generation transplanted murine tumour models. *Br J Cancer* **77**: 1752–1760

Robinson SP, Collingridge DR, Howe FA, Rodrigues LM, Chaplin DJ and Griffiths JR (1999) Tumour response to hypercapnia and hyperoxia monitored by FLOOD magnetic resonance imaging. *NMR Biomed* **12**: 98–106

Sansom JM and Wood PJ (1994) $^{31}$P MRS of tumour metabolism in anaesthetized vs conscious mice. *NMR Biomed* **7**: 167–171

Sauer LA, Stayman JW and Dauchy RT (1982) Amino acid, glucose and lactic acid utilization in vivo by rat tumors. *Cancer Res* **42**: 4090–4097

Song CW (1998) Modification of blood flow. In: *Blood Perfusion and Microenvironment of Human Tumors*, Molls M and Vaupel P (eds), pp. 193–207. Springer-Verlag: Berlin, Heidelberg

Stubbs M, Robinson SP, Rodrigues LM, Parkins CS, Collingridge DR and Griffiths JR (1998) The effects of host carbogen (95% $O_2$/5% $CO_2$) breathing on metabolic characteristics of Morris hepatoma 9618a. *Br J Cancer* **78**: 1449–1456

Thomlinson RH and Gray LH (1955) The histological structure of some human lung cancers and the possible implications for radiotherapy. *Br J Cancer* **9**: 539–549

Tozer GM and Griffiths JR (1992) The contribution made by cell death and oxygenation to $^{31}$P MRS observations of tumour energy metabolism. *NMR Biomed* **5**: 279–289

van den Boogaart A, Howe FA, Rodrigues LM, Stubbs M and Griffiths JR (1995) In vivo $^{31}$P MRS: absolute concentrations, signal-to-noise and prior knowledge. *NMR Biomed* **8**: 87–93

Vaupel P, Kallinowski F and Okunieff P (1989) Blood flow, oxygen and nutrient supply, and metabolic microenvironment of human tumors: a review. *Cancer Res* **49**: 6449–6465

Williams SNO, Rajagopolan B, Adams GE, Hayes D and Brindle KM (1996) A comparative study of tumor vascularization and blood flow in spontaneous and transplanted tumors. *Proc Intl Soc Magn Reson Med* **2**: 1109

Wood PJ, Counsell CJR, Bremner JCM, Horsman MR and Adams GE (1991) The measurement of radiosensitizer-induced changes in mouse tumor metabolism by $^{31}$P magnetic resonance spectroscopy. *Int J Radiat Oncol Biol Phys* **20**: 291–294

Yamaura H and Matsuzawa T (1979) Tumor regrowth after irradiation. An experimental approach. *Int J Radiat Biol* **35**: 201–219

*© 2000 Cancer Research Campaign*
